# Biological Control of the Plant Pathogens

**DOI:** 10.3390/microorganisms11122930

**Published:** 2023-12-06

**Authors:** Rainer Borriss, Chetan Keswani

**Affiliations:** 1 Institute of Biology, Humboldt University, 10115 Berlin, Germany; 2 Institute of Marine Biotechnology e.V., 17489 Greifswald, Germany; 3 Academy of Biology and Biotechnology, Southern Federal University, 44090 Rostov-on-Don, Russia

(This article belongs to the Special Issue **Biological Control of the Plant Pathogens.**)

The ultimate effects of crop losses manifest in the form of insufficient food production and chronic hunger. The gravity of this issue is rapidly being amplified by the rises in urbanization, climate change, and emerging pests and pathogens, and the deterioration of soil health. As an environmentally friendly alternative to chemical pesticides, microbial biocontrol agents (BCAs) have attracted global attention due to their ability to ensure food security by directly halting pre-harvest crop losses and, thereby, improving crop productivity [1]. Despite the substantial progress achieved in our understanding of plant microbe interactions in recent years, developing more efficient BCAs remains a constant task. The current advances in biocontrol science and technology and the avenues for future research are reflected in the contributions presented in this Special Issue. These can be categorized as follows:

(i) *Management strategies for major soilborne pathogens in crops*: Ma et al. (contribution 1) review tomato production systems and their challenges. He et al. (contribution 2) have optimized *Metarhizium robertsii* fermentation broth for the efficient management of wolfberry root rot. Fuller et al. (contribution 3) author an informative review on the beneficial and pathogenic microbiota associated with the common alder (*Alnus glutinosa*).
(ii) *Biocontrol potential of novel volatile compounds and defense inducers*: Recent research has revealed that, besides antimicrobial peptides (AMPs), other natural compounds are involved in biocontrol action. Two examples are given in this Special Issue. Hernández et al. (contribution 4) characterize 65 potential VOCs of the *Kosakonia cowanii* Cp1 strain and demonstrate their role in the effective biocontrol of various economically important phytopathogens. Tripathi et al. (contribution 5) explore defense inducers, including salicylic acid, isonicotinic acid, benzothiadiazole, and lysozymes, as prophylactic and curative sprays for inducing resistance in tomato plants against *Clavibacter michiganensis* subsp. *michiganensis*.
(iii) *Exploring the biocontrol potential of novel microbial isolates and genetically engineered strains*: *Bacillus velezensis* FZB42 is known as a prototype for Gram-positive plant-beneficial bacteria, able to produce durable endospores and suppress plant pathogens [2]. The potential of a novel isolate of *B. velezensis,* strain BV01, as a broad-spectrum BCA against various fungal phytopathogens was assessed (contribution 6). In addition to *B. velezensis*, other representatives of the Bacillaceae family, isolated from Vietnamese crop plants, contain a rich spectrum of compounds efficient against plant pathogens. Numerous members of the *B. cereus* species complex have been comprehensively investigated for the occurrence of the biosynthetic gene clusters (BGCs) involved in the synthesis of secondary metabolites. Antimicrobial peptides, efficient against pathogenic fungi and nematodes, and entomocidal crystal proteins have been detected and partially characterized using mass spectrometry (contribution 7). Moreover, genome mining in plant-associated *Brevibacilli* and *Lysinibacillus* spp. has revealed 36 novel BGCs, not present in the MIBiG data bank, which could be developed as next-generation biocontrol agents (contribution 8). Novel fungal isolates, efficient against grapevine phytopathogens, are described (contribution 9). Interestingly, fungal strains isolated from overwintered tar spot stromata could serve as potential BCAs against tar spot disease in corn (contribution 10). Cui et al. (contribution 11) have engineered an *Escherichia coli* strain to express jasmonic acid carboxyl methyl transferase that catalyzes the conversion from jasmonic acid to methyl jasmonate and demonstrate its biocontrol potential in the management of fungal smut disease.
(iv) *Formulation technology*: Lin et al. (contribution 12) comment on the recent progress in developing “green” adjuvants used for formulating long-living BCAs compatible with chemical pesticides.

Together, the articles published in this Special Issue give an insight into recent and future trends in the development of more powerful and reliable BCAs, and will contribute to sustainable agriculture.
